# Simultaneous Spectrophotometric Estimation of Imipramine Hydrochloride and Chlordiazepoxide in Tablets

**DOI:** 10.4103/0250-474X.57304

**Published:** 2009

**Authors:** Sejal Patel, N. J. Patel, S. A. Patel

**Affiliations:** S. K. Patel College of Pharmaceutical Education and Research, Department of Pharmaceutical Chemistry, Ganpat University, Kherva, Mehsana-382 711, India

**Keywords:** Imipramine HCl, chlordiazepoxide, simultaneous equation method, Q-absorption ratio method, first derivative spectrophotometry

## Abstract

A binary mixture of imipramine HCl and chlordiazepoxide was determined by three different spectrophotometric methods. The first method involved determination of imipramine HCl and chlordiazepoxide using the simultaneous equations and the second method involved absorbance ratio method. Imipramine has absorbance maxima at 251 nm, chlordiazepoxide has absorbance maxima at 264.5 nm and isoabsorptive point is at 220 nm in methanol. Linearity was obtained in the concentration ranges of 1-25 and 1-10 μg/ml for Imipramine HCL and Chlordiazepoxide, respectively. The third method involved determination of these two drugs using the first-derivative spectrophotometric technique at 219 and 231.5 nm over the concentration ranges of 1-20 and 2-24 μg/ml with mean accuracies 99.46±0.78 and 101.43±1.20%, respectively. These methods were successively applied to pharmaceutical formulations because no interferences from the tablet excipients were found. The suitability of these methods for the quantitative determination of the compounds was proved by validation.

Imipramine HCl (IMI) is chemically, (10,11-Dihydro-N,N-dimethyl)-5H-dibenz[b,f] azepine-5-propanamine[1]. It is a tricyclic antidepressant used in case of depression[2]. Chlordiazepoxide (CLR) is chemically, 7-chloro-N-methyl-5-phenyl-3H-1,4-benzodiazepin-2-amine 4-oxide[1]. It is an anxiolytic agent but a poor anticonvulsant[2]. IMI is official in IP, BP and USP. The IP[3], BP[4] and USP[5] describe non-aqueous titration, potentiometric titration and HPLC methods, respectively for estimation of IMI. A literature survey revealed comparison of HPLC and fluorescence polarization immunoassay method[6], determination by UV spectrophotometric method[7] drug dissolution study[8] and HPLC/DAD screening method[9] of IMI with other antipsychotic agents like nortriptyline, amitriptyline. CLR is official in IP, BP and USP. The IP[3], BP[4] and USP[5] describe non-aqueous titration, potentiometric titration and HPLC methods, respectively for the estimation of CLR. Literature survey revealed determination of major impurity of CLR by an UV method[10] and HPLC method with FTIR and UV detection[11] in formulations. Literature survey also reported spectrophotometric[12], difference spectrophotometric[13], micellar liquid chromatography[14] and derivative spectrophotometry[15] methods for CLR with other drugs in pharmaceutical formulations. IMI and CLR are formulated together in the form of a tablet. The purpose of this study was to determine both drugs concurrently by simple, accurate, rapid and precise simultaneous equation, Q-absorbance ratio and first derivative spectrophotometric assays for routine analysis.

All absorption spectra and derivatives were recorded with a UV-1700 PC UV/Vis double beam spectrophotometer with spectral width of 2 nm, wavelength accuracy of 0.5 nm and a pair of 10 mm matched quartz cells (Shimadzu, Japan). CP224S analytical balance (Sartorius) and ultra sonic cleaner (Frontline FS 4) were used throughout the study. Standard samples of IMI and CLR were generous gifts from Torrent Pharmaceuticals Ltd. (Ahmedabad, India). Marketed tablets of Libomin tablets (Consern Pharma), each containing 25 mg IMI and 10 mg CLR were used. Methanol (S. D. Fine Chemical, Ahmedabad, India) used was of pure analytical grade.

IMI and CLR stock solutions (0.5 mg/ml, each) were prepared by weighing accurately 50 mg each powder into 2 separate 100 ml volumetric flasks, 50 ml methanol was added, shaken for a few minutes and diluted to volume with methanol. These stock solutions (2 ml) were transferred into 2 separate 10 ml measuring flasks and diluted to the mark with methanol to a final concentration of 100 μg/ml each.

For the simultaneous equation method, working standard solutions having concentrations 1, 5, 10, 15, 20 and 25 μg/ml for IMI and 1, 2, 4, 6, 8 and 10 μg/ml for CLR were prepared in methanol using the stock solutions. Working standard solutions were scanned in the entire UV range of 200-400 nm to determine the λ-max of both drugs. The λ-max of IMI and CLR were found to be 251 nm and 264.5 nm, respectively. The absorbances of the working standard solutions were measured at 251 nm and 264.5 nm and calibration curves were plotted at these wavelengths. The absorptivity coefficients of these two drugs were determined using calibration curve equations.

For Q-absorbance ratio method uses the ratio of absorbances at two-selected wavelengths one at isoabsorptive point and other being the λ-max of one of the two components. From the overlay spectra of two drugs, it was evident that IMI and CLR show an isoabsorptive point at 220 nm. The second wavelength used was 264.5 nm, which is the λ-max of CLR. Working standard solutions having concentration 1, 5, 10, 15, 20 and 25 μg/ml for IMI and 1, 2, 4, 6, 8 and 10 μg/ml for CLR were prepared in methanol using stock solutions and the absorbances at 220 nm (isoabsorptive point) and 264.5 nm, (λ-max of CLR) were measured and absorptivity coefficients were calculated using calibration curve.

For the first derivative spectrophotometric method, accurate aliquots of IMI equivalent to 1-20 μg/ml were transferred from its stock solution (100 μg/ml) in to a series of 10 ml volumetric flasks and diluted to mark with methanol and mixed well. Accurate aliquots of CLR equivalent to 2-24 μg/ml were transferred from its working solution (100 μg/ml) in to a series of 10 ml volumetric flasks and diluted to mark with methanol and mixed well. Considering all the derivative order spectra of IMI and CLR from first to fourth derivative, the first derivative order spectra with d (N) =2 was found suitable. The zero crossing point on the first derivative spectra of one drug, the other drug shows substantial absorbance, these two wavelengths can be employed for the estimation of IMI and CLR without any interference from other drug in combined formulations. From the derivatised spectra of prepared mixtures the absorbances were measured at 219 nm for IMI and 231.5 nm for CLR. These absorbances Vs concentration were plotted in the quantitative mode to obtain the working curves from which by extrapolating the value of absorbances of the sample solution, the concentration of the corresponding drugs were determined. Both the drugs obeyed Beer's Law.

Powder from the mixed contents of 20 tablets, equivalent to 25 mg IMI and 10 mg CLR, was transferred accurately to a 50 ml volumetric flask and diluted to volume with methanol. The solution was diluted to the same concentrations of working standard solutions and treated according to the linearity for the different spectrophotometric methods.

This work is devoted to the analysis of IMI and CLR, which are available together in the form of tablets. By reviewing the literature concerning the determination of IMI and CLR in mixtures, it was found that no method was reported for simultaneous determination of the two drugs. Therefore, the aim of this work was to develop simple analytical methods for the simultaneous determination of IMI and CLR. This was achieved using simultaneous equation, Q- absorbance ratio and first derivative spectrophotometric methods.

For the simultaneous equation method, two wavelengths of respective absorbance maxima i.e. 251 nm for IMI and 264.5 nm for CLR were used for the analysis of the drugs. Fig. 1 shows simple overlain spectra of IMI and CLR. The criteria for obtaining maximum precision[16], by this method were calculated and found to be out side the range of 0.1-2. Two simultaneous Eqns were formed. A1= 432×(Cx+1260)×y and A2= 331×(Cx+1349)×Cy, where Cx and Cy are concentrations of IMI and CLR, respectively in g/100 ml in the sample solution. A1 and A2 are the absorbances of the mixture at 251 nm and 264.5 nm, respectively. The concentration of Cx and Cy can be obtained as Cx= [(A2×1260)−(A1×1349)]/-165708 and Cy= [(A1×331)−(A2×432))]/-165708.

**Fig. 1:**
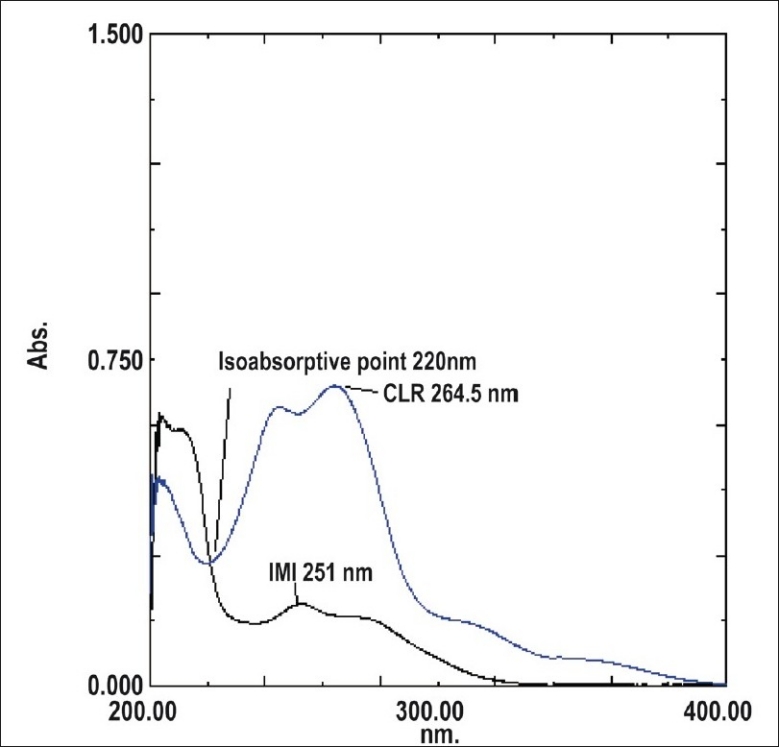
Overlain spectra of IMI and CLR. IMI is imipramine HCl and CLR is chlordiazepoxide.

For the Q-absorbance ratio method, two wavelengths of 220 nm (isoabsorptive point) and 264.5 nm (λ-max of CLR) were used for the analysis of the drugs. Fig. 1 shows overlain spectra of IMI and CLR showing isoabsorptive point. The concentration of two drugs in the mixture can be calculated using Eqns, Cx= Q_M_-Q_Y_/Q_X_-Q_Y_×A_1_/ax_1_ and C_Y_ = A_1_/ax_1_−Cx, where Cx and Cy are concentrations of IMI and CLR, respectively in g/100 ml in the sample solution. A_1_ is the absorbances of the mixture at 220 nm. ax_1_ and ay_1_ are absorptivity of IMI and CLR respectively at 220 nm; ax_2_ and ay_2_ are absorptivity of IMI and CLR respectively at 264.5 nm and Q_M_ = A_2_/A_1_, Q_X_ = ax_2_/ax_1_ and Q_Y_ = ay_2_/ay_1_.

First derivative spectrophotometric method is used to eliminate the spectral interference from one of the two drugs while estimating the other drug by selecting the zero crossing point on the derivative spectra of each drug as the selected wavelength. Fig. 2 shows overlain first derivative spectra of IMI and CLR. IMI can be assayed in the presence of CLR by measuring absorption at zero crossing point of CLR in the range of 1-20 μg/ml. The linear regression Eqn was found to be: Y=0.006X+0.0063, r= 0.9984, where Y is the absorbance value at 219 nm, X is the concentration in μg/ml, and r is the correlation coefficient. CLR can be assayed in the presence of IMI by measuring absorption at zero crossing point of IMI in the range of 2-24 μg/ml. The linear regression Eqn was found to be: Y=0.0031X+0.0016, r= 0.9995, where Y is the absorbance value at 231.5 nm, X is the concentration in μg/ml, and r is the correlation coefficient.

**Fig. 2:**
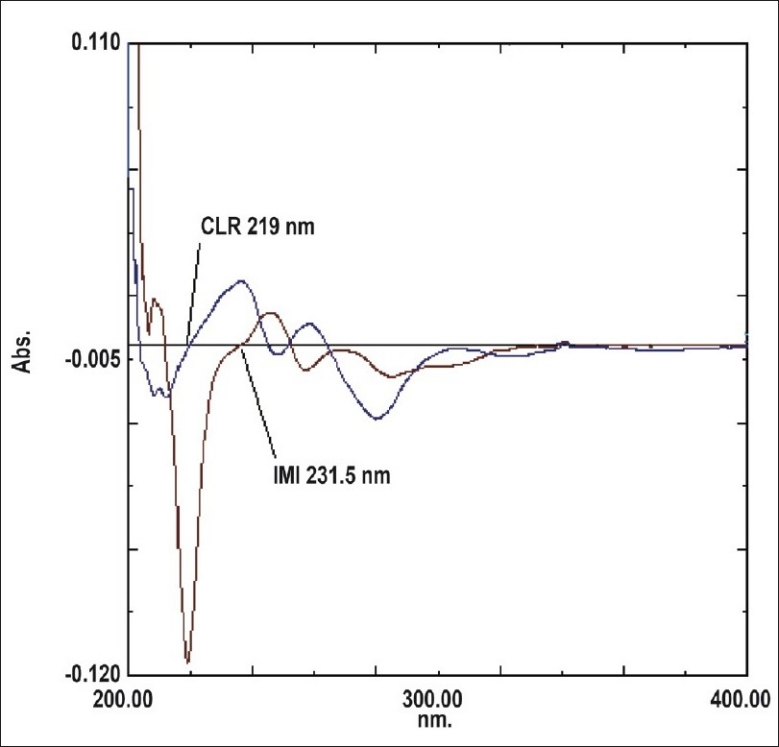
First derivative absorption spectra of IMI and CLR. IMI is imipramine HCl and CLR is chlordiazepoxide

The validity of the suggested procedures was further assessed by applying the standard addition techniques (Table 1). The proposed methods have been applied to assay IMI and CLR in tablets without any interference from the additives (Table 2). The results of assay validation of the proposed methods show that they are accurate and precise according to the RSD values of intraday and interday determinations (Table 3).

**TABLE 1:** APPLICATION OF THE STANDARD ADDITION TECHNIQUE TO THE ANALYSIS OF IMI AND CLR IN TABLETS BY THE PROPOSED METHODS

Proposed methods	Concentration of drug taken (μg/ml)		Concentration of drug added (μg/ml)		Concentration of drug found (μg/ml)		% Recovery (n^a^=3) ± SD^b^	
				
	IMI	CLR	IMI	CLR	IMI	CLR	IMI	CLR
Simultaneous equation	5	2	2.5	1	7.57	2.98	100.9±0.93	99.3±1.34
	5	2	5	2	10.18	3.94	101.8±1.0	98.5±0.76
	5	2	7.5	3	12.66	4.95	101.2±1.38	99±0.90
Q- absorbance ratio	5	2	2.5	1	7.64	2.97	101.8±1	99±1.33
	5	2	5	2	10.02	3.99	100.2±1.05	99.7±0.75
	5	2	7.5	3	12.65	4.99	101.2±1.45	99.8±0.8
First derivative UV	5	2	2.5	1	7.45	3.03	99.3±0.98	101±1.2
	5	2	5	2	9.95	4.05	99.5±0.65	101.3±1.37
	5	2	7.5	3	12.45	5.1	99.6±0.72	102±1.05

a n is the number of determinations

b SD is standard deviation, IMI is imipramine HCl and CLR is chlordiazepoxide

**TABLE 2:** ASSAY RESULTS FOR TABLETS USING THE PROPOSED METHODS

Formulation	Proposed methods	Mix.	Amount of drug added (mg)		Amount of drug found (mg)		% Amount found (n^a^=3) ± SD^b^	
					
			IMI	CLR	IMI	CLR	IMI	CLR
Tablets	Simultaneous	1	25	10	24.72	10.1	98.9±0.95	101±1.14
	equation	2	25	10	24.8	10.07	99.2±1.05	100.8±1.1
	Q-absorbance ratio	1	25	10	24.78	10.15	99.1±0.9	101.5±1.12
		2	25	10	24.82	10.12	99.31	101.3±1.14
	First derivative UV	1	25	10	24.98	10.3	99.9±0.61	102.8±1.52
		2	25	10	25.16	9.98	100.7±0.7	99.8±1.11

a n is the number of determinations

b SD is standard deviation, IMI is imipramine HCl and CLR is chlordiazepoxide

**TABLE 3:** SUMMARY OF VALIDATION PARAMETERS FOR THE PROPOSED METHODS

Proposed methods	Drug	Parameters			
					
		LOD^a^ μg/ml	LOQ^b^ μg/ml	Interday (*n*=3) (RSD^c^, %)	Intraday (n^d^=3) (RSD c, %)
Simultaneous equation	IMI (251 nm)	0.313	0.95	0.82-2.32	0.60-1.81
	IMI (264.5 nm)	0.326	0.99	0.70-2.86	0.62-1.59
	CLR (251 nm)	0.214	0.65	1.05-2.58	0.94-2.17
	CLR (264.5 nm)	0.191	0.579	0.88-2.81	0.44-1.87
Q-absorbance ratio	IMI (264.5 nm)	0.303	0.92	0.69-2.95	0.69-1.56
	CLR (264.5 nm)	0.245	1.742	0.88-2.81	0.44-1.61
	IMI+CLR (220 nm)	0.291	0.882	0.78-3.75	0.68-2.69
First derivative UV	IMI (219 nm)	0.313	0.95	1.14-2.89	1.04-2.79
	CLR (231.5 nm)	0.59	1.8	1.49-2.91	1.50-3.53

a LOD is limit of detection

b LOQ is limit of quantification

c RSD is relative standard deviation and

d n is the number of determinations, IMI is imipramine HCl and CLR is chlordiazepoxide

These methods were compared by applying the analysis of variance (ANOVA) test. The calculated F-value of 7.25 for IMI and 0.92 for CLR are less than the tabulated F-value (9.55) at the 95% confidence interval, which reveals that there is no significant difference with respect to accuracy and precision between the proposed methods. The proposed procedures can be applied for the simultaneous determination of IMI and CLR. Moreover, the methods are rapid, accurate, precise and can be used for routine analysis.
